# Salinity-Induced Physiological Changes in Pea (*Pisum sativum* L.): Germination Rate, Biomass Accumulation, Relative Water Content, Seedling Vigor and Salt Tolerance Index

**DOI:** 10.3390/plants11243493

**Published:** 2022-12-13

**Authors:** Mohammad Ayub Hossain Khan, Md. Abdul Baset Mia, Md. Abdul Quddus, Khokan Kumer Sarker, Mohibur Rahman, Milan Skalicky, Marian Brestic, Ahmed Gaber, Amnah Mohammed Alsuhaibani, Akbar Hossain

**Affiliations:** 1 Regional Agricultural Research Station, Bangladesh Agricultural Research Institute, Cumilla 3500, Bangladesh; 2 Department of Crop Botany, Bangabandhu Sheikh Mujibur Rahman Agricultural University, Gazipur 1706, Bangladesh; 3 Soil and Water Management Section, Horticulture Research Centre, Bangladesh Agricultural Research Institute (BARI), Gazipur 1701, Bangladesh; 4 Department of Botany and Plant Physiology, Faculty of Agrobiology, Food, and Natural Resources, Czech University of Life Sciences Prague, Kamycka 129, 165 00 Prague, Czech Republic; 5 Institute of Plant and Environmental Sciences, Slovak University of Agriculture, Nitra, Tr. A. Hlinku 2, 949 01 Nitra, Slovakia; 6 Department of Biology, College of Science, Taif University, P.O. Box 11099, Taif 21944, Saudi Arabia; 7 Department of Physical Sport Science, College of Education, Princess Nourah bint Abdulrahman University, P.O. Box 84428, Riyadh 11671, Saudi Arabia; 8 Soil Science Division, Bangladesh Wheat and Maize Research Institute, Dinajpur 5200, Bangladesh

**Keywords:** salinity, germination, seedling vigor, field pea, physiological response

## Abstract

Salinity affects and limits the yield potential of pulse crops. Therefore, an experiment was conducted to evaluate the salinity-induced physiological response of field peas by estimating the germination rate (%), accumulation of biomass, relative water content, and seedling vigor and salt tolerance index. The treatments included four salinity levels (NaCl) (i.e., 0 (control), 8, 12, and 16 dS m^−1^, respectively) and eight field pea genotypes (i.e., BD4175, BD4182, BD4225, BD6944, BD4176, BD4193, BD4493, and BD4496). All treatments were arranged in a factorial completely randomized design and repeated four times. Results indicated that the percentage and rate of germination, percentage reduction of fresh and dry weight, relative water content, seedling vigor index, and salt tolerant index of all genotypes of field peas were influenced significantly by the different levels of salinity. The radicle and plumule of all field pea genotypes were damaged by applying 12 and 16 dS m^−1^ salt stress. However, among these eight pea genotypes, two genotypes, namely BD4175 and BD4225, performed better under the 8 dS m^−1^ level of salinity and these two genotypes may be recommended for cultivation in field conditions of saline coastal areas of Bangladesh, and can also be used in future breeding programs for the development of salt-tolerant pea cultivars.

## 1. Introduction

Field pea (*Pisum sativum* L.) is a crucial winter vegetable legume crop of the *Fabaceae* family [1] widely grown in Southwest Asia, Europe, North America, Japan, Australia, Mediterranean countries, and Ethiopia [2] for versatile uses, viz., dry pulses, fresh peas, and fodder for cattle [1,3]. As shown in [1,2], it is high in minerals (Fe, Mg, P, and Zn), vitamins (A, C, K, thiamine, niacin, folic acid, pyridoxine, and pantothenic acid), carbs, and protein (19–27%) but low in antinutritional compounds [4,5]. Due to its low fat, salt, and cholesterol levels, field pea prevents cardiovascular diseases [6]. It has been recognized as a soil fertility restorer due to its symbiotic nitrogen fixation capacity [3,7].

Various biotic and abiotic stresses drastically reduce the production of this high-value crop. Amongst abiotic stresses, salinity is extremely devastating, which hampers crop growth, development, and yield severely [8,9,10,11]. Salinity refers to a build-up of soluble salts [12,13,14]. Salinity affects a lot of land around the world, and it is worsening every day. This is making irrigated crop fields really difficult to manage [15]. Globally, at least 20% of total irrigated lands are salt-affected [16]. Salinity has been identified as a critical issue globally, since it is predicted to have an impact on 30% of the world’s cultivated land area during the next 25 years and around 50% of the land area by the end of this century. [17].

On the other hand, salt stress hampers global food security and food balance [18]. Salinity affected areas of Bangladesh are located in the coastal region. The coastal region covers almost 29,000 square kilometers, of which about 53% are affected by salinity [11,12,13,14,15,16,17,18,19]. 

In comparison to soybean and fava bean, pea is more sensitive to salinity [20]. Field pea yields were shown to be reduced by almost 50% at 100 mM NaCl [21], and productivity decreased after being exposed to high salinity levels [22]. Salt stress impacts plant metabolism by lowering potential water, ion imbalance and toxicity, and CO_2_ absorption in arid and semi-arid settings, where it is also the main factor limiting legume production [23,24,25,26]. A decrease in photosynthesis and crop growth is caused by salinity (Na^+^), which interferes with K^+^ and Ca^2+^ nutrition and disrupts the effective regulation of stomata. Salinity (Na^+^) interferes with K^+^ and Ca^2+^ nutrition and disturbs efficient stomata regulation, resulting in a depression of photosynthesis and crop growth. Cl^−^ toxicity also reduces the photosynthetic capacity, ultimately decreasing crop growth and yield [27,28]. Salt stress decreases the germination percentage and rate, causes delayed seed germination, and hampers the vigor of seed, root, and shoot growth or seedling growth of crops, such as legume and senna (*Cassia angustifolia)* crops [29,30]. Crop species and cultivars respond differently to saline soils in terms of germination and seedling growth [31]. Additionally, the type of plant, the level of salt, and the toxicity of the ions all affect growth and yield reduction [32,33]. Therefore, depending on the plant species, salt stress has varying effects [34]. Seed germination in salt stress is one of the most critical and viable stages in establishing seedlings [35] for selecting the salt-tolerant field pea genotype in a screening trial with eight cultivars. The majority of horticultural and legume crops are extremely vulnerable to saline stress during the germination and seedling stages. There has not been a thorough investigation of how salinity affects the morphological characteristics of seedlings and the evaluation of germination in field pea genotypes.

In light of these opinions, the current study was carried out to determine the optimal salinity level and suitable genotypes of field pea under salinity stress circumstances, as well as to evaluate the physiological response of field pea to salinity by evaluating germination rate, biomass accumulation, relative water content, seedling vigor, and salt tolerance index.

## 2. Results

### 2.1. Germination Percentage

**Figure 1:**
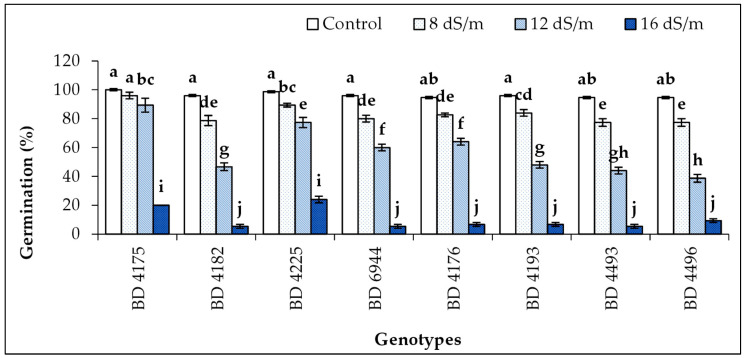
Effect of salt stress on the rate of germination (%) of selected field pea genotypes under laboratory conditions; vertical bars indicate the standard error, mean values of above bars with similar letters are not significantly different using least significant difference (LSD) test at *p* ≤ 0.05.

The germination percentage of all field pea genotypes was significantly influenced by the application of different salinity stresses (Figure 1). In the study, the germination percentage exhibited a decreasing trend with the increase in salinity level. The highest and comparable germination percentage was found in all genotypes under control conditions, and the lowest was in 16 dS m^−1^ salt stress condition. The highest germination percentage (100%) was observed in genotype BD 4175; the second highest (99%) was in BD 4225; the lowest was in BD 4182, BD 6944, and BD 4493.

### 2.2. Germination Rate 

**Figure 2:**
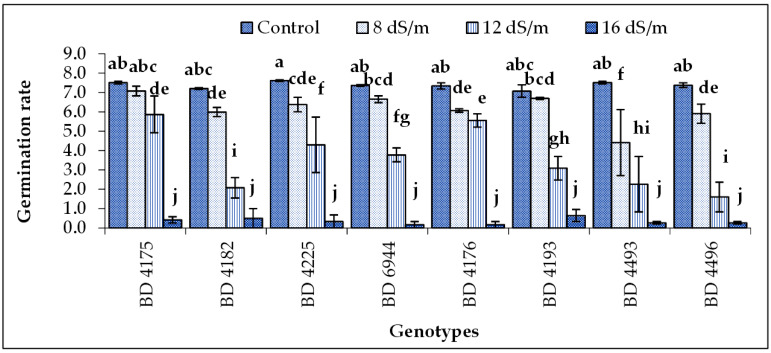
Effect of salt stress on the germination rate of selected field pea genotypes under laboratory conditions; vertical bars indicate the standard error, mean values of above bars with similar letters are not significantly different using LSD test at *p* ≤ 0.05.

The highest germination rate was recorded under control conditions for all genotypes but was statistically similar among genotypes. It decreased with increasing salinity (Figure 2). Among the genotypes, the maximum germination rate was recorded in genotype BD 4225 followed by BD 4175 and BD 4493 under control conditions. The lowest was found in BD 4496 followed by BD 4182 at 12 dS m^−1^ salt stress condition. All genotypes’ germination rate was negligible in 16 dS m^−1^ salt stress conditions. 

### 2.3. Relative Water Content

**Figure 3:**
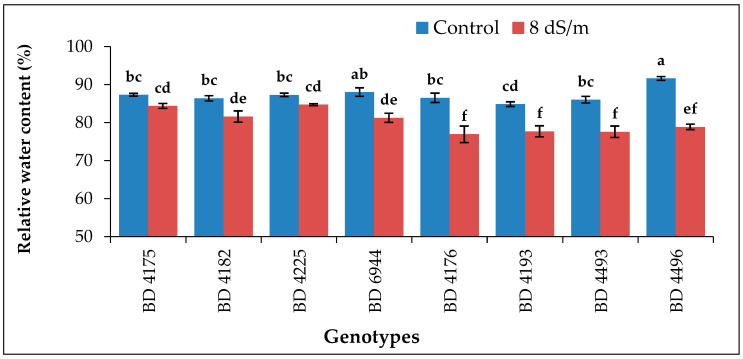
Effect of salt stress on the relative water content of selected field pea genotypes under laboratory conditions; vertical bar represents the standard error, mean values of above bars with similar letters are not significantly different using LSD test at *p* ≤ 0.05.

Salinity, imposed in terms of different concentrations, positively affected the relative water content in the genotypes. Increasing salinity triggered the reduction of water content in leaf tissue. Significant differences in relative water content were exposed in leaf tissue among genotypes at different NaCl levels (Figure 3). Regarding the genotypes, the highest relative water content (91.59%) was recorded from BD 4496, comparable with BD 6944 under the control condition and the lowest (84.84%) was from BD 4193. In the case of 8 dS m^−1^ salt stress, the highest relative water content value (84.70%) was noted from BD 4225 followed by BD 4175 (84.38%) among genotypes, and the lowest (76.92%) was from BD 4176. The relative water content value was not found in all genotypes at salinity stress 12 dS m^−1^ and 16 dS m^−1^ because the radicle and plumule of genotypes were rotten.

### 2.4. Shoot Length

**Figure 4:**
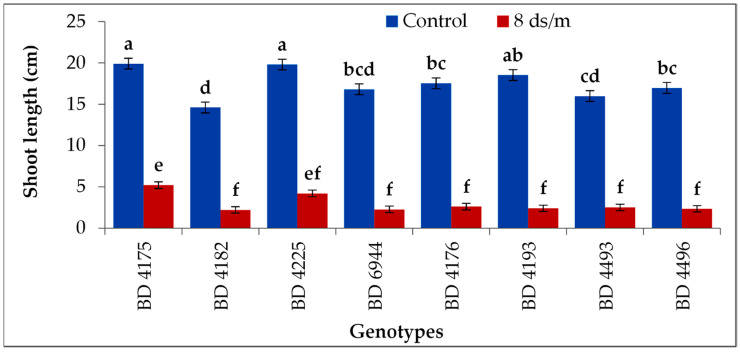
Effect of salt stress on shoot length of selected field pea genotypes under laboratory conditions; vertical bar represents the standard error, mean values of above bars with similar letters are not significantly different using LSD test at *p* ≤ 0.05.

Salinity affected the shoot length (plumule length) for all genotypes (Figure 4). The highest shoot length (19.9 cm in control and 5.2 cm in salt stress 8 dS m^−1^) was achieved in BD 4175 which was statistically identical to BD 4225. The lowest shoot length was in BD 4182, which was on par with BD 6944, BD 4496, BD 4493, BD 4193, and BD 4176.

### 2.5. Root Length

**Figure 5:**
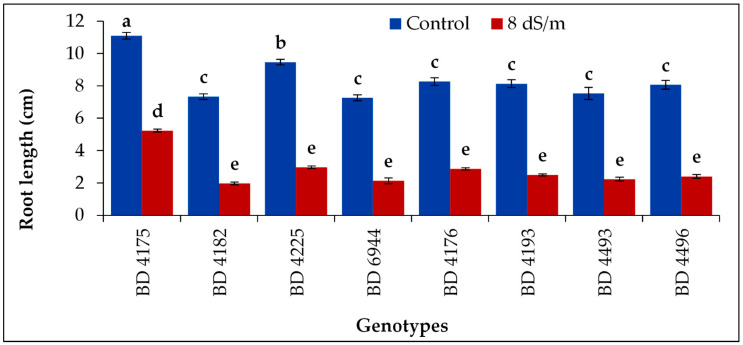
Effect of salt stress on root length of selected field pea genotypes under laboratory conditions; vertical bar represents the standard error, mean values of above bars with similar letters are not significantly different using LSD test at *p* ≤ 0.05.

The root length (radicle length) of all field pea genotypes was decreased due to the application of NaCl. Significantly, the highest root length (11.1 cm in control and 5.23 cm in salt stress 8 dS m^−1^) was recorded from genotype BD 4175, and the lowest (1.97 cm) was from BD 4182 under 8 dS m^−1^ salt stress condition (Figure 5). 

### 2.6. Percentage Reduction of Fresh Weight

**Figure 6:**
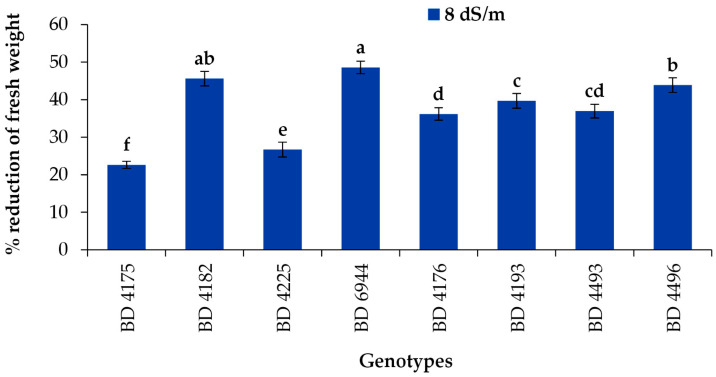
Effect of salt stress (8 dS m^−1^) on fresh weight percentage reduction of field pea genotypes under laboratory conditions; vertical bar represents the standard error, mean values of above bars with similar letters are not significantly different using LSD test at *p* ≤ 0.05.

The fresh weight percentage reduction varied from 22.6% to 48.6% among the genotypes of field pea due to the application of salt stress 8 dS m^−1^ (Figure 6). However, significantly, the highest fresh weight percentage reduction was observed in genotype BD 6944 followed by BD 4182, and the lowest reduction was found in BD 4175.

### 2.7. Percentage Reduction of Dry Weight 

**Figure 7:**
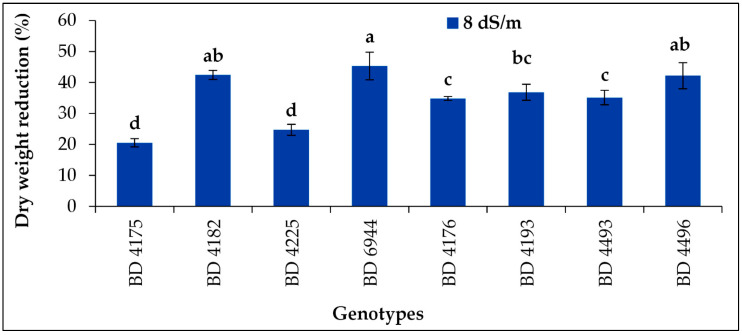
Effect of salt stress (8 dS m^−1^) on dry weight percentage reduction of selected field pea genotypes under laboratory conditions; vertical bar represents the standard error, mean values of above bars with similar letters are not significantly different using LSD test at *p* ≤ 0.05.

The dry weight percentage reduction (DWPR) ranged from 20.5% to 45.3% among the genotypes under the salt stress 8 dS m^−1^ (Figure 7). However, the highest DWPR was obtained from BD 6944 comparable with BD 4182 and BD 4496. The lowest reduction was from BD 4175 followed by BD 4225. The shoot and root dry weight was decreased with increasing the level of salt stress. 

### 2.8. Seedling Vigor Index

**Figure 8:**
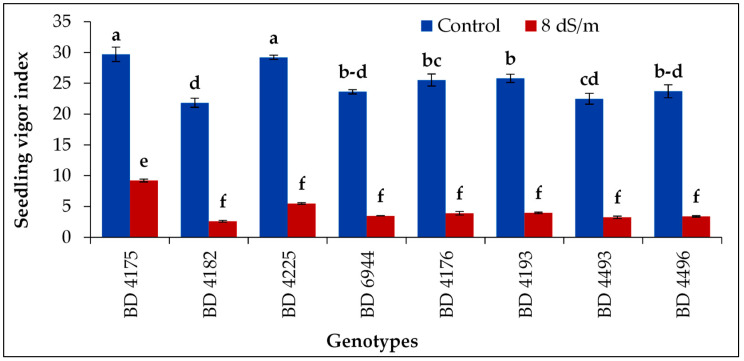
Effect of salt stress (8 dS m^−1^) on seedling vigor index of selected field pea genotypes under laboratory conditions; vertical bar represents a standard error, mean values of above bars with similar letters are not significantly different using LSD test at *p* ≤ 0.05.

Salinity stress affected the seedling vigor index of all field pea genotypes. Significantly the highest seedling vigor index under salt stress condition of 8 dS m^−1^ was achieved in genotype BD 4175, followed by BD 4225, and the lowest index was found in BD 4182 genotype (Figure 8). The decrease in seedling vigor index with increasing salinity indicates that salt content has a negative impact on seeds. 

### 2.9. Salt Tolerant Index

**Figure 9:**
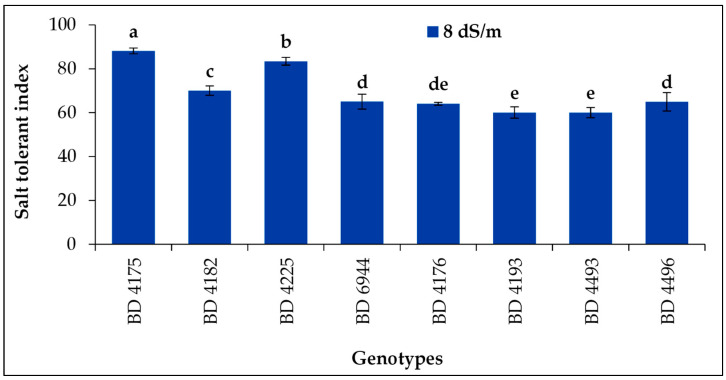
Effect of salt stress on salt (8 dS m^−1^) tolerant index of selected field pea under laboratory conditions; here, the vertical bar represents the standard error, and mean values of the above bars with similar letters are not significantly different using LSD test at *p* ≤ 0.05.

Salinity stress 8 dS m^−1^ affected the salt tolerance index (STI) of all genotypes of field pea shown in Figure 9. Significantly, the highest value of STI was recorded from the genotype BD 4175, and the lowest value of STI was from genotypes BD 4193 and BD 4493. The STI decreased with increasing salinity stress.

### 2.10. Seedling Height Stress Index

**Figure 10:**
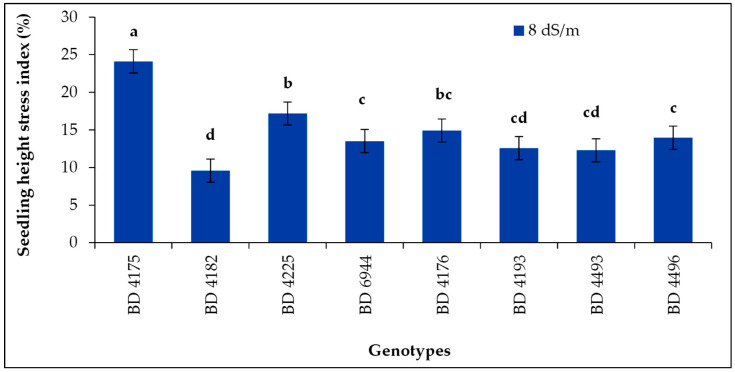
Effect of salt stress (8 dS m^−1^) on seedling height stress index of selected field pea under laboratory conditions; vertical bar represents the standard error, mean values of above bars with similar letters are not significantly different using LSD test at *p* ≤ 0.05.

The seedling height stress index (SHSI) of all genotypes was influenced significantly by salt stress 8 dS m^−1^ (Figure 10). Significantly, the highest value of SHSI (24.1%) was noted from the genotype BD 4175, and the lowest value of SHSI was from the genotype BD 4182 followed by BD 4193 and BD 4493 under salt stress 8 dS m^−1^. The seedling height stress index was decreased with the increasing salinity stress. Most of the genotypes exhibited their tolerance at germination or seedling growth stage at salinity level 8 dS m^−1^.

## 3. Discussion

Salinity creates adverse environmental conditions limiting seed germination and crop production [36,37]. However, the results of the present study have been emphasized to screen out the potential field pea genotype under different salt stress levels and evaluate the appropriate salinity level. The abiotic stress salinity hampered seed germination and seedling growth, eventually limiting crop production [15,24]. The germination rate was highest in control for all the genotypes, but it declined in increased salinity levels because boosted salt concentrations reduce the germination rate [38]. Among the genotypes, BD4225, BD 4175, and BD 4493 exhibited the highest germination rate, and the lowest germination rate was observed in BD 4182 and BD 4496 at 8 and 12 dS m^−1^ salinity levels, respectively. The germination rate was very negligible at 16 dS m^−1^ salt stress. Awasthi et al. [14] found a similar outcome, stating that Vigna species’ seed germination rates decreased as a result of increased salt stress. Depending on the plant species, salt stress impacts germination percentage, germination rate, and seedling growth in various ways [24,35]. It has been suggested that osmotic stress and specific ion toxicity, which are caused by aberrant ions interacting through Na^+^ and Cl^−^, are to blame for the detrimental effects of salt stress on seed germination [39,40]. Sodium chloride (NaCl) can have a negative impact on the embryo’s development, which may therefore negatively impact seed germination [41]. When the ions Na^+^ and Cl^−^ enter plant cells, the tolerant plants can classify them in the vacuole or the cytoplasm for sensitive cultivars [42]. Additionally, NaCl might prevent some enzymes from functioning properly, which could be crucial for seed germination [41]. The toxic effects of salt reduce the water potential in the medium, which hinders seed germination by preventing water absorption [43]; this results in a decrease in the germination rate.

The relative water content of leaf tissue is its present water content relative to its maximum water content at full turgidity. The RWC is related to water uptake by the roots as well as water loss through transpiration. Salinity, imposed in terms of different concentration levels, profoundly affected the relative water content in all genotypes used in the present experiment. The highest relative water content was found in BD 4496, and the second highest was in BD 6944, which showed the minimum variations among other genotypes in control. The stress condition (8 dS m^−1^) for the genotypes BD 4175 and BD 4225 exhibited the maximum relative water content. Reduced water content was found in stress conditions of 12 dS m^−1^ and 16 dS m^−1^. Similarly, reduced water contents with increased salt stress were reported by several researchers in different crops, such as rice and *Medicago polymorpha* [44,45], while root moisture contents were enhanced with increasing salinity levels. 

Salinity significantly influenced the shoot length (plumule length) in the genotypes tested; however, the most extended shoot (plumule) was achieved by the genotype BD 4175, and the shortest shoot (plumule) was observed in BD 4182. During the early phases of seedling development, an increase in salt content results in a decrease in growth because salt has osmotic potential [46]. The toxic effect of Na^+^ ions, which build up in leaves, becomes obvious in the later stages and slows plant growth. Additionally, under salt stress, shoot development may be inhibited due to a reduced supply of essential nutrients [47]. The length of the shoot and root are decreased with increasing salt concentration levels in legumes, such as clover, as observed by various scientists [48]. Root length decreased due to the addition of NaCl; however, the highest root length was achieved in BD 4175, and the lowest was obtained from BD 4182 in salt stress conditions. The adverse effect of the salt stress also showed the differences in the magnitude concerning chloride ions. Increased levels of salt in the growth media can inhibit cell division by reducing osmotic potential and reducing water absorption [49]. Our findings also showed that plumule and radicle length were decreasing, which had an impact on cell division. The length of the plumule and radicle reduction is caused by the saline effect [50]. Other reports stated that this reduction may also be caused by ionic toxicity, disruption in nutrient intake, and osmotic impact on plant water absorption, which would reduce the biosynthesis of enzymes and plant hormones that are required for growth [51]. 

A significant percentage reduction of fresh weight was found due to the application of salt stress in selected field pea genotypes. However, the highest reduction was observed in BD 6944, followed by BD 4182, and the lowest reduction was observed in BD 4175. The outcome is consistent with Stoeva and Kaymakanova’s findings [52], who noted a sharp decline in the fresh shoot weight of leguminous plant seedlings in a salinity-induced environment. Since metabolic synthesis occurs in the leaves and is severely disrupted under high salt stress, either due to the reduced water uptake or the toxic effect of NaCl concentration, this reduction may be caused by the limited availability of metabolites to early growing tissues [53,54]. The results of the present investigation were consistent with those of Bayuelo-Jiménez et al. [55] and Kagan et al. [56], who found that fresh root weight was considerably decreased by salinity rise in *Phaseolus* species and *Lens culinaris*, respectively. Our findings were supported by earlier studies on *Lens culinaris, Triticum aestivum,* and *sorghum bicolor* [57,58]. The seedlings’ unbalanced nutrient uptake, as well as the toxic effects of the increased level of NaCl concentration, may be the reason for reduced root and shoot development. NaCl toxicity and an imbalance in seedling nutrient uptake may be responsible for the decline in root and shoot growth. The fresh and dry weights of the shoot and root were reduced because of salt stress. Additionally, the shoot or root lengths had a significant impact on this reduction [57,58]. Salt stress considerably reduced the plant’s fresh and dry weight in *Physalis* species [59]. Different salt levels significantly affected growth parameters by lowering the biomass and length of the root and shoot [60].

Similar trends to those in fresh weight percentage loss are seen in dry weight percentage reduction. However, BD 4225 and BD 6944 had the biggest reductions in dry weight percentage, whereas BD 4175 and BD 4182 had the lowest reductions. With increased salt stress, the weights of the shoots and dry roots dropped. Similar outcomes were acquired in spider plants (*Cleome gynandra* L.) [61] and the white seed coat *Bambara* groundnut (*Vigna subterranean* L. verdc.) landrace with high-salt treatment (200 mM NaCl) [62]. The current result is consistent with Al-Mutawa’s [63] observations on chickpeas, who noted that high saline concentrations reduced the dry weights of the impacted crops. The vigor index of the seedlings decreased as salinity levels rose, indicating that increased salt concentrations had a negative impact on the vigor index and seed germination percentage of common beans (*Phaseolus vulgaris* L.) [64]. Additionally, the maize seedling vigor index was considerably impacted by various salt stressors [65]. As reported previously [66], under stressful circumstances, water absorption decreases both during imbibition and seedling establishment. Under salt stress, this could be followed by an increase in ion uptake [66]. Additionally, it has been noted that salt inhibits the absorption of vital nutrients like P and K, which may have a negative impact on seedling growth and vigor index [67].

Increasing salinity stress resulted in a decline in the salt tolerance index. Similar results were reported by Awasthi et al. [14]. *Vigna mungo* displayed the maximum STI at 50 mM salt stress level (46.5%), whereas *Vigna radiata* displayed the lowest STI at 100 mM salt stress level (20.8%) [14]. When the salinity was increased to 12 dS m^−1^ and 16 dS m^−1^, the higher seedling height stress index in BD 4175 was no longer present. This result is consistent with that of Al-Mutawa [63], who discovered that high saline concentrations decreased the chickpea (*Cicer arietinum* L.) genotype’s seedling height stress index.

## 4. Materials and Methods

### 4.1. Experimental Site, Treatments, and Design

The experimental study was carried out in a lab setting at the Department of Crop Botany, Bangabandhu Sheikh Mujibur Rahman Agricultural University, Gazipur, Bangladesh. The ambient temperature at the laboratory varied from 12 to 32.0 °C, relative humidity averaged 82.5%, and the average light and dark photoperiod hours were similar by 12 h during the study period. The 32 treatment combinations comprised four levels of salinity and eight field pea genotypes. The salinity levels were 0 (control), 8, 12, and 16 dS m^−1^, and the field pea genotypes were BD 4175, BD 4182, BD 4225, BD 6944, BD 4176, BD 4193, BD 4493, and BD 4496. All of the listed genotypes’ seeds were acquired from The Plant Genetic Resources Center of Bangladesh Agricultural Research Institute, Gazipur. Four replications of a factorial randomized design (CRD) were used to set up the experiment. 

### 4.2. Experimental Procedures

In order to create 1 dS m^−1^ of salinity, commercial salt (NaCl) was dissolved in distilled water at a rate of 640 mg L^−1^. The amount of salinity in the solution was determined using an electrical conductivity tester (HI-98303 EC Meter). To keep the salinity levels consistent, 5 mL of saline solution was added daily to each Petri plate [59]. Each genotype’s seeds were surface sterilized with 5% sodium hypochlorite (NaOCl) for 5 min [68], then washed by rinsing in distilled water. The surface sterilized seeds were placed in Petri dishes that contained double-layered Whatman filter paper No. 1. Each experiment consisted of 32 Petri dishes and was repeated four times. All of the 128 Petri plates were cleaned in tap water, rinsed with distilled water, and then sterilized at 170 °C for 4 h in a hot air sterilizer Digital Autoclave Machine (Sterilizer SS-V100HD, Labtex Bangladesh, Dhaka, Bangladesh) [69]. In each Petri dish, 50 seeds of each genotype were distributed across two layers of Whatman paper. The control Petri dish contained 10 mL of distilled water, and the treatment Petri dish contained 10 mL of salt solution. The Petri dishes were monitored every day for up to 18 days, and 10 mL of distilled water for the control and 10 mL of a salt solution for each treatment were added to the Petri dishes every day. Daily counts of seeds that germinated were carried out after their emergence in each Petri dish for up to 18 days. A seed was considered to have germinated when the plumule protrusions were 2 mm long. Eighteen days after the seedlings first appeared, they were collected, and for each treatment, the shoot length, root length, and fresh and dry weight were measured. The final count of germination percentage (GP) and germination rate (GR) was documented after germination.

### 4.3. Rate of Germination

According to the seedling evaluation process outlined in the Association of Official Seed Analysis [70], the germination percentage (GP) was calculated by counting the number of seeds that germinated each day. The GP was calculated ten days after germination by dividing the number of seeds that germinated in any Petri dishes by the total number of seeds tested:

(1) $$\mathrm{GP}\;\left(\%\right)=\frac{\mathrm{Number}\;\mathrm{of}\;\mathrm{total}\;\mathrm{germinated}\;\mathrm{seeds}}{\mathrm{The}\;\mathrm{total}\;\mathrm{number}\;\mathrm{of}\;\mathrm{seeds}\;\mathrm{tested}}\times 100$$

The germination rate (GR) was determined according to the following formula [7]:

(2) $$\mathrm{GR}=\frac{\mathrm{Number}\;\mathrm{of}\;\mathrm{germinated}\;\mathrm{seeds}}{\mathrm{Day}\;\mathrm{of}\;\mathrm{the}\;\mathrm{first}\;\mathrm{count}}+\ldots \;\ldots \;\ldots +\frac{\mathrm{Number}\;\mathrm{of}\;\mathrm{germinated}\;\mathrm{seeds}}{\mathrm{Day}\;\mathrm{of}\;\mathrm{the}\;\mathrm{final}\;\mathrm{count}}$$

### 4.4. Fresh and Dry Weight

After 48 h of oven drying at 70 °C in the Digisystem Laboratory Digital Hot Air Oven (DSO-500D), root and shoot dry weights were determined. The fresh and dry weights specified as the controls were computed as percentages based on each salt treatment using the following equations [71]:

FWPR (%) = [1 − (Fresh weight salt stress/fresh weight control)] × 100(3)

DWPR (%) = [1 − (dry weight salt stress/dry weight control)] × 100(4)

FWPR and DWPR are the fresh and dry weight percentage reduction, respectively.

### 4.5. Estimation of Several Physiological Parameters

The following measurements were performed: the seedling vigor index (SVI), relative water content (RWC), salt tolerance index (STI), and seedling height stress index (SHSI). The seedlings with primary roots that were too short were thought to have germinated abnormally [72]. When the radicle length reached 10 mm, a field pea seed was considered to have germinated [73].

#### 4.5.1. Seedling Vigor Index

The seedling vigor index (SVI) was calculated as the product of GP and the average value of seedling length (shoot + root) [74] and expressed by the following equation:

(5) $$\mathrm{SVI}=\frac{\mathrm{GP}\;\times \;\mathrm{seedling}\;\mathrm{length}\;}{100}$$

#### 4.5.2. Salt Tolerance Index

The following equation is used to determine the salt tolerance index (STI), which is measured as a ratio to the total dry weight under salt stress and represented as a percentage:

(6) $$\mathrm{STI}\;\left(\%\right)=\frac{\mathrm{DW}\;\left(\mathrm{salt}\;\mathrm{stress}\right)}{\mathrm{DW}\;\left(\mathrm{control}\right)}\times 100$$

#### 4.5.3. Relative Water Content

The amount of water in leaf tissue compared to its maximum water content at full turgidity is known as relative water content, or RWC. Water absorbed by roots and water lost through transpiration is influenced by the RWC. The following formula was used to determine the RWC [75].

(7) $$\mathrm{RWC}\;\left(\%\right)=\frac{\mathrm{FW}\;-\;\mathrm{DW}}{\mathrm{TW}\;-\;\mathrm{FW}}\times 100$$

To calculate the RWC, the third leaf of each chosen plant which was considered as the youngest fully developed leaf was used. As soon as the leaf was detached, it was weighed, and the result was recorded as fresh weight (FW). The cut end of the entire leaf was inserted into a test tube filled with distilled water, closed with cotton wool, and stored in a light environment in the lab. The leaves were removed after five hours, blotted dry, and re-weighed to determine their turgid weight (TW); they were then dried at 80 °C for twenty-four hours and reweighed to determine their dry weight (DW).

#### 4.5.4. Plumule and Radicle Length

The length of the embryo’s plumule, expressed in centimeters, was measured and recorded. The distance in centimeters between the first cotyledonary node’s point and the tip of the longest radicle was determined.

#### 4.5.5. Seedling Length Stress Index

The seedling length stress index (SLSI) was calculated using the following formula [63].

(8) $$\mathrm{SLSI}\;\left(\%\right)=\frac{\mathrm{Seedling}\;\mathrm{length}\;\mathrm{of}\;\mathrm{stressed}\;\mathrm{seedling}}{\mathrm{Seedling}\;\mathrm{length}\;\mathrm{of}\;\mathrm{the}\;\mathrm{controlled}\;\mathrm{seedling}}\times 100$$

### 4.6. Statistical Analysis

The experiment was set up using a completely random design (CRD) with two parameters (salinity and genotypes). The statistical software Statistix-10 [76] was used to carry out a two-way analysis of variance. The LSD test was used to determine whether two comparisons were significantly different when the *p*-value was ≤0.05.

## 5. Conclusions

According to the findings of the current investigation, the genotypes showed significant differences in their ability to adjust to salt stress. Salinity has a big effect on all different types of growth parameters. Among salt stress, including the control, only control and 8 dS m^−1^ salt stresses exhibited better seed germination of all genotypes, and seedlings were raised better. The other salinity levels, viz. 12 and 16 dS m^−1,^ when applied to the seeds, some seeds germinated, but after one week of seed germination, the radicle and plumule were damaged for all of the genotypes. Germination percentage and rate, fresh and dry weight percentage reduction, relative water content, plumule length, radicle length, seedling vigor index, salt tolerant index, and seedling length stress index were significantly influenced by the induction of salt stress. Among the genotypes, BD 4175 and BD 4225 performed better under salt stress of 8 dS m^−1^ and could be an alternate option for sustainable field pea cultivation in the field conditions of saline areas.

## Data Availability

Not applicable.

## References

[1] Shahid S., Shahbaz M., Maqsood M.F., Farhat F., Zulfiqar U., Javed T., Fraz Ali M., Alhomrani M., Alamri A.S. (2022). Proline-Induced Modifications inMorpho-Physiological, Biochemical and Yield Attributes of Pea (*Pisum sativum* L.) Cultivars under Salt Stress. Sustainability.

[2] Kanwar P.S., Toppo S., Sahu S. (2020). Evaluate the performance of genotypes of pea in terms of growth, yield and quality attributes. J. Pharmacogn. Phytochem..

[3] Quddus M.A., Hossain M.A., Naser H.M., Anwar B., Akhtar S., Nazimuddin M. (2018). Effect of zinc and boron application on productivity, quality and nutrient uptake of fieldpea (*Pisum sativum* L.) grown in calcareous soils. J. Agric. Sci. Pract..

[4] Sofy M.R., Elhindi K.M., Farouk S., Alotaibi M.A. (2020). Zinc and Paclobutrazol Mediated Regulation of Growth, Upregulating Antioxidant Aptitude and Plant Productivity of Pea Plants under Salinity. Plants.

[5] Dahl W.J., Foster L.M., Tyler R.T. (2012). Review of the health benefits of peas (*Pisum sativum* L.). Br. J. Nutr..

[6] Verma A.K., Banerjee R., Sharma B.D. (2015). Quality characteristics of low fat chicken nuggets: Effect of salt substitute blend and pea hull flour. J. Food Sci. Technol..

[7] Hussain F., Islam M., Zaman A. (2006). Ethnobotanical profile of plants of Shawar Valley, District Swat, Pakistan. Int. J. Biol. Biomol. Agric. Food Biotechnol. Eng..

[8] Nusrat N., Shahbaz M., Perveen S. (2014). Modulation in Growth, Photosynthetic Efficiency, Activity of Antioxidants and Mineral Ions by Foliar Application of Glycinebetaine on Pea (*Pisum sativum* L.) under Salt Stress. Acta Physiol. Plant..

[9] Siringam K., Juntawong N., Cha-um S., Boriboonkaset T., Kirdmanee C. (2012). Salt tolerance enhancement in *indica* rice (*Oryza sativa* L. spp. *indica*) seedlings using exogenous sucrose supplementation. Plant Omics J..

[10] Bilashini Devi M., Thoithoi Devi M., Jha A.K., Anjoo Y., Balusamy A., Verma V.K., Talang H.D., Deshmukh N.A., Rymbai H., Assumi S.R. (2018). Yield and Yield Attributes of Garden Pea (*Pisum sativum* var. *hortense* L.) as Influenced by Nutrient Management Practices under Agroclimatic Conditions of Meghalaya. Int. J. Curr. Microbiol. App Sci..

[11] Bartels D., Sunkar R. (2005). Water and salt tolerance in plants. Crit. Rev. Plant. Sci..

[12] Alharbi K., Al-Osaimi A.A., Alghamdi B.A. (2022). Sodium Chloride (NaCl)-Induced Physiological Alteration and Oxidative Stress Generation in *Pisum sativum* (L.): A Toxicity Assessment. ACS Omega.

[13] Ibraheem O., Dealtry G., Roux S., Bradley G. (2011). The effect of drought and salinity on the expressional level of sucrose transporters in rice (*Oryza sativa* Nipponbare) cultivar plants. Plant Omics J..

[14] Awasthi P., Karki H., Vibhuti, Bargali K., Bargali S.S. (2016). Germination and Seedling Growth of Pulse Crop (*Vigna* spp.) as Affected by Soil Salt Stress. Curr. Agric. Res. J..

[15] Shahid M.A., Pervez M.A., Balal R.M., Abbas T., Ayyub C.M., Mattson N.S., Riaz A., Iqbal Z. (2012). Screening of pea (*Pisum sativum* L.) genotypes for salt tolerance based on early growth stage attributes and leaf inorganic osmolytes. Aust. J. Crop. Sci..

[16] Ahmad F., Kamal A., Singh A., Ashfaque F., Alamri S., Siddiqui M.H. (2022). Salicylic Acid Modulates Antioxidant System, Defense Metabolites, and Expression of Salt Transporter Genes in Pisum sativum Under Salinity Stress. J. Plant Growth Regul..

[17] Goumi Y.E., Fakiri M., Lamsaouri O., Benchekroun M. (2011). Salt stress effect on seed germination and some physiological traits in three Moroccan barley (*Hordeum vulgare* L.) cultivars. Ann. Biol. Res..

[18] Lam Y., Winch P.J., Nizame F.A., Broaddus-Shea E.T., Harun M., Dostogir G., Surkan P.J. (2022). Salinity and food security in southwest coastal Bangladesh: Impacts on household food production and strategies for adaptation. Food Sec..

[19] Nenova V. (2008). Growth and mineral concentrations of pea plants under different salinity levels and iron supply. Gen. Appl. Plant Physiol..

[20] Subbarao G.V., Nam N.H., Chauhan Y.S., Johansen C. (1994). Osmotic adjustment, water relations and carbohydrate remobilization in pigeon pea under water deficits. J. Plant Physiol..

[21] Ouerghi K., Abdi N., Maazaoui H., Hmissi I., Bouraoui M., Sifi B. (2016). Physiological and morphological characteristics of pea (*Pisum sativum* L.) seeds under salt stress. J. New Sci. Agric. Biotechnol..

[22] Najafi F., Khavari-Nejad R.A., Rastgar-Jazii F., Sticklen M. (2007). Growth and some physiological attributes of pea (*Pisum sativum* L.) as affected by salinity. Pak. J. Biol. Sci..

[23] Rozema J., Flowers T. (2008). Crops for a salinized world. Science.

[24] Lluch C., Tejera N., Herrera-Cervera J.A., Lopez M., Barranco-Gresa J.R., Palma F.J., Ocana A. (2007). Saline stress tolerance in legumes. Lotus News Lett..

[25] Abdel Latef A. (2010). Changes of antioxidative enzymes in salinity tolerance among different wheat cultivars. Cereal Res. Commun..

[26] Munns R., James R.A. (2003). Screening methods for salinity tolerance: A case study with tetraploid wheat. Plant Soil.

[27] Møller I.S., Gilliham M., Jha D., Mayo G.M., Roy S.J., Coates J.C., Haseloff J., Tester M. (2009). Shoot Na^+^ exclusion and increased salinity tolerance engineered by cell type-specific alteration of Na^+^ transport in Arabidopsis. Plant Cell.

[28] Ullah M.A., Aamir S.S., Haider H., Adil B., Mahmood I.A., Badar-uz-Zaman, Hyder S.I. (2018). Effect of salinity, humic acid, biozote and vermicompost on soil physicochemical properties and olive plants species. J. Agric. Sci. Pract..

[29] Sharma J.R. (2006). Statistical & Biometrical Techniques in Plant Breeding.

[30] Shitole S.M., Dhumal K.N. (2012). Effect of water stress by polyethylene glycol 6000 and sodium chloride on seed germination and seedling growth of *Cassia angustifolia*. Int. J. Pharm. Sci. Res..

[31] Rahim T., Tlili I., Hnan I., Ilahy R., Ben A.A., Jabbari H. (2013). Effect of stress on saline compartment physiological metabolic trials varieties of pigments (*Capsicum annum* L.). J. Appl. Biosci..

[32] Tavakkoli E., Rengasamy P., McDonald G.K. (2011). High concentrations of Na+ and Cl^−^ ions in soil solution have simultaneous detrimental effects on growth of faba bean under salinity stress. J. Exp. Bot..

[33] Yadav S.S., McNeil D.L., Redden R., Patil S.A. (2010). Climate Change and Management of Cool Season Grain Legume Crops.

[34] Ayed S., Rassaa N., Chamekh Z., Beji S., Karoui F., Bouzayen T., Mrabat M., Ben Y.M. (2014). Effect of salt stress (sodium chloride) on germination and seedling growth of durum wheat (*Triticum durum* Desf.) genotypes. Int. J. Biodivers. Conversat..

[35] Ungar I.A. (1996). Effect of salinity on seed germination, growth, and ion accumulation of *Atriplexpatula* (Chenopodiaceae). Am. J. Bot..

[36] Shrivastava P., Kumer R. (2015). Soil salinity: A serious environmental issue and plant growth promoting bacteria as one of the tools for its alleviation. Saudi J. Biol. Sci..

[37] Haque S.A. (2006). Salinity problems and crop production in coastal regions of Bangladesh. Pak. J. Bot..

[38] Cicek N., Cakirlar H. (2002). The effect of salinity on some physiological parameters in two maize cultivars. Bulg. J. Plant Physiol..

[39] Faheed F.A., Hassanein A.M., Azooz M.M. (2005). Gradual increase in NaCl concentration overcomes inhibition of seed germination due to salinity stress in *Sorghum bicolor* (L.). Acta Agron. Hung..

[40] Jovičić D., Nikolić Z., Zorić M., Marjanović-Jeromela A., Petrović G., Milošević D., Ignjatov M. (2014). Viability of oilseed rape (*Brassica napus* L.) Seeds under salt stress. Genetika.

[41] Grozeva S., Kalapchieva S., Tringovska I. (2019). Evaluation of garden pea cultivars to salt stress tolerance. Mech. Agric. Conserv. Resour..

[42] Akhtar M., Hussain F., Ashraf M.Y., Qureshi T.M., Akhter J., Awan A.R. (2012). Influence of salinity on nitrogen transformations in soil. Commun. Soil Sci. Plant Anal..

[43] Farsiani A., Ghobadi M.E. (2009). Effects of PEG and NaCl stress on two cultivars of corn (*Zea mays* L.) at germination and early seedling stages. Int. J. Biol. Biomol. Agric. Food Biotechnol. Eng..

[44] Polash M.A.S., Sakil M.A., Tahjib-Ul-Arif M., Hossain M.A. (2018). Effect of salinity on osmolytes and relative water content of selected rice genotypes. Trop. Plant Res..

[45] Ibrar M., Hussain F. (2003). The effect of salinity on the growth of *Medicago polymorpha* Linn. J. Sci. Technol. (Peshawar).

[46] Munns R., Tester M. (2008). Mechanisms of salinity tolerance. Annu. Rev. Plant Biol..

[47] Dagar J.C., Bhagwan H., Kumar Y. (2004). Effect on growth performance and biochemical contents of *Salvadora persica* when irrigated with water of different salinity. Indian J. Plant Physiol..

[48] Jajarmi V. (2009). Effect of water stress on germination indices in seven wheat cultivar. World Acad. Sci. Eng. Technol..

[49] Ashraf M., Harris P. (2005). Abiotic Stresses: Plant Resistance through Breeding and Molecular Approaches.

[50] Kausir Z., Mariem B.F., Fardaous M., Cherif H. (2012). Impact of salt stress (NaCl) on growth, chlorophyll content and fluorescence of Tunisian cultivars of chili pepper (*Capsicum frutescens* L.). J. Stress Physiol. Biochem..

[51] Bor M., Özdemir F., Türkan I. (2003). The effect of salt stress on lipid peroxidation and antioxidants in leaves of sugar beet *Beta vulgaris* L. and wild beet *Beta maritima* L.. Plant Sci..

[52] Stoeva N., Kaymakanova M. (2008). Effect of Salt Stress on the Growth and Photosynthesis Rate of Bean Plants (*Phaseolus vulgaris* L.). J. Cent. Eur. Agric..

[53] Hussain K., Majeed A., Nawaz K., Nisar M.F. (2009). Effect of different levels of salinity on growth and ion contents of black seeds (*Nigella sativa* L.). Curr. Res. J. Biol. Sci..

[54] Taffouo V.D., Kouamou J.K., Ngalangue L.M.T., Ndjeudji B.A.N., Akoa A. (2009). Effects of salinity stress on growth, ions partitioning and yield of some cowpea (*Vigna unguiculata* L. Walp.) cultivars. Int. J. Bot..

[55] Bayuelo-Jiménez J.S., Craig R., Iynch J.P. (2002). Salinity Tolerance of *Phaseolus* species during Germination and Early Seedling Growth. Crop Sci..

[56] Kagan K., Karakoy T., Bakoglu A., Akçura M. (2010). Determination of salinity tolerance of some lentil (*Lens culinaris* M.) varieties. J. Food Agric. Environ..

[57] Akbarimoghaddam H., Galavi M., Ghanbari A., Panjehkeh N. (2011). Salinity effects on seed germination and seedling growth of bread wheat cultivars. Trakia J. Sci..

[58] Naim A.M., Mohammed K.E., Ibrahim E.A., Suleiman N.N. (2012). Impact of salinity on seed germination and early seedling growth of three sorghum (*Sorghum biolor* L. Moench) cultivars. Sci. Technol..

[59] Yildirim E., Dursun A., Güvenc I., Kumlay A.M. (2000). The effects of different salt, biostimulants and temperature levels on seed germination of some vegetable species. II Balkan Symposium on Vegetables and Potatoes.

[60] Datta J.K., Nag S., Banerjee A., Mondai N.K. (2009). Impact of salt stress on five varieties of wheat (*Triticum aestivum* L.) cultivars under laboratory condition. J. Appl. Sci. Environ. Manag..

[61] Mwai G.N. (2001). Growth Response of Spiderplant (*Cleome gynandra* L.) to Salinity. Ph.D. Dissertation.

[62] Taffouo V.D., Wamba O.F., Youmbi E., Nono G.V., Akoa A. (2010). Growth, yield, water status and ionic distribution response of three bambara groundnut (*Vigna subterranean* (L.) verdc.) landraces grown under saline conditions. Int. J. Bot..

[63] Al-Mutawa M.M. (2003). Effect of salinity on germination and seedling growth of chickpea (*Cicer arietinum* L.) genotypes. Int. J. Agric. Biol..

[64] Cokkizgin A. (2012). Salinity stress in common bean (*Phaseolus vulgaris* L.) seed germination. Not. Bot. Horti Agrobot. Cluj-Napoca.

[65] Janmohammadi M., Dezfuli P.M., Sharifzadeh F. (2008). Seed invigoration techniques to improve germination and early growth of inbred line of maize under salinity and drought stress. Gen. Appl. Plant Physiol..

[66] Prisco J.T., Vieira G.H.F. (1976). Effects of NaCl salinity on nitrogenous compounds and proteases during germination of *Vigna* sinensis seeds. Physiol. Plant..

[67] Nasim M., Qureshi R., Aziz T., Saqib M., Nawaz S., Sahi S.T., Pervaiz S. (2008). Growth and ionic composition of salt stressed *Eucalyptus camaldulensis* and *Eucalyptus teretcornis*. Pak. J. Bot..

[68] Younesikelaki F.S., Ebrahimzadeh M.H., Desfardi M.K., Banala M., Marka R., Nanna R.S. (2016). Optimization of seed surface sterilization method and in vitro seed germination in *althaea oicinalis* (L.)-An important medicinal herb. Indian J. Sci. Technol..

[69] Muhammad Z., Hussain F. (2010). Effect of NaCl salinity on the germination and seedling growth of some medicinal plants. Pak. J. Bot..

[70] Association of Official Seed Analysis (AOSA) (1990). Rules for testing seeds. J. Seed Technol..

[71] Vibhuti, Shahi C., Bargali K., Bargali S.S. (2015). Assessment of salt stress tolerance in three varieties of rice (*Oryza sativa* L.). J. Progress. Agric..

[72] ISTA (2011). Rules Proposals for the International Rules for Seed Testing. International Seed Testing Association.53p. Secretariat, Zürichstrasse 50, CH-8303 Bassersd of Switzerland. https://www.seedtest.org/en/publications/international-rules-seed-testing.html.

[73] Goertz S.H., Coons J.M. (1989). Germination response of tepary and navy beans to sodium chloride temperature. Hortscience.

[74] Abdul-Baki A.A., Anderson J.D. (1973). Vigour determination in soybean seed by multiple criteria. Crop. Sci..

[75] Sumithra K., Jutur P.P., Carmel B.D., Reddy A.R. (2006). Salinity induced changes in two cultivars of *Vigna radiata*: Responses of antioxidative and proline metabolism. Plant Grow. Regul..

[76] Statistix 10 (1985). An Analytical Software.

